# Small skin incision and less suture material reduce granuloma after laparoscopic gastrostomy: a prospective observational study in children

**DOI:** 10.1007/s00383-026-06347-0

**Published:** 2026-02-23

**Authors:** Therese Hössjer, Niclas Högberg, Johan Danielson

**Affiliations:** Department of Pediatric Surgery, Institution of Women’s and Children’s Health, Akademiska Sjukhuset, Uppsala University, Akademiska Barnsjukhuset, 751 85 Uppsala, Sweden

**Keywords:** Pediatric surgery, Laparoscopic gastrostomy, Postoperative minor complications, Tube feeding, Granuloma

## Abstract

**Purpose:**

Gastrostomy serves as an essential device for children with insufficient oral intake. Laparoscopic techniques are increasingly used and considered safe, although minor complications remain common. The purpose of this prospective observational study was to compare three different laparoscopic gastrostomy techniques and evaluate the incidence of complications at three months postoperatively.

**Methods:**

Children < 18 years of age scheduled for gastrostomy at participating centers between 2014 and 2019 were included. Data on demographics, perioperative details, and three-month postoperative complications were collected. Three laparoscopic techniques were compared: Method A (intracorporeal double U-stitch), Method B (mini-laparotomy with fascial and stomach purse-string sutures), and Method C (extracorporeal double U-stitch combined purse-string suture).

**Results:**

A total of 310 patients (median age: 27.0 months, median weight: 10.9 kg) from three centers were included. Three months postoperatively, the incidence of granuloma was significantly higher in Method B (89.7%) compared to Method A (59.3%) and Method C (35.2%) (*p* < 0.001). Method C had the lowest rates of granuloma, infection, leakage, and pain, while dislocation rates were similar across methods.

**Conclusion:**

Our findings suggest that a surgical technique with a small skin incision and less suture material positioned away from the gastrostomy site may help reduce postoperative complications, particularly granuloma.

## Introduction

Gastrostomy serves as an essential device for children with various disabilities, and is one of the most common gastrointestinal procedures in pediatric in-patient care. Placement of a gastrostomy is indicated when long-term enteral nutrition is required and there is an insufficient oral intake [1]. The majority of children requiring a gastrostomy belong to a vulnerable patient group that requires frequent health care visits, which challenge both families and society. Therefore, researchers emphasize the need for improvements in the care process to enhance outcomes for both families and the health care system [2, 3].

Over the years, in-patient care has improved alongside the evolvement of operative techniques. The transition from open surgery and PEG (percutaneous endoscopic gastrostomy) has been followed by the development of several different and combined approaches, including laparoscopy, endoscopy and radiologically assisted methods [4]. The use of laparoscopic techniques is now considered the safest approach by several researchers, and has increased over time [5–15]. The adoption of less invasive techniques has improved the initial postoperative course, leading to reduced pain and a shorter hospital stay [16]. However, despite these advancements, minor postoperative complications remain common, affecting 62–73% of all children receiving a gastrostomy [17–19]. The complications that arise often persists for a long time, requiring repeated medical visits and treatments.

Various laparoscopic methods have been described [20–22], and a few comparative studies suggest that different laparoscopic techniques can lead to varying rates of complications [19, 23–27]. A systematic review on gastrostomy tube use in children identified three key areas for improvement in the care of these patients, one of which is optimizing the placement technique to minimize postoperative complications [3].

The aim of this nationwide study was to compare three different laparoscopically assisted techniques and assess the incidence of minor complications three months postoperatively.

## Methods

### Study design and data collection

Data was collected between 2014 and 2019 as a nationwide non-randomized prospective study. Before inclusion, oral and written information about the study was provided, and each family gave their written informed consent. The principles of the Declaration of Helsinki were followed, and the study was approved by the Regional Committee on Medical Research Ethics in Uppsala (Dnr 2014/219 and Dnr 2018/486).

The study collected in total 86 different variables from four non validated questionnaires. Following surgery, the surgeon completed the initial questionnaire, which gathered details on the operative technique, duration, gastrostomy size and type, antibiotic use, any concomitant procedures, as well as patient characteristics and demographics. At the routine three-month follow-up, a specialist nurse practitioner (or, in a few cases, a doctor) completed the second questionnaire. This questionnaire collected data on postoperative complications—including granuloma; pain (defined as pain at the gastrostomy site experienced by patients or families); dislocation; leakage; and infection (including all patients treated with oral or topical antibiotics)—as well as information on complication management, gastrostomy function, healing, cosmetic outcomes, and any reoperations performed. Two additional questionnaires, administered at three and twelve months postoperatively, were similarly designed for families to provide their perspectives on these complications, treatment strategies, gastrostomy function, and cosmesis.

In this study, we aim to compare the three different laparoscopic techniques and evaluate the incidence of postoperative complications. Since the operative site is expected to be healed within three months, we have chosen to analyze data exclusively from the three-months follow-up.

This paper does not include an analysis of cosmesis and function. Additionally, we have previously published a study focusing on complications in relation to the gastrostomy size (thickness and length in relation to the gastrostomy canal), indication for gastrostomy, operative technique and the use of antibiotic prophylaxis [17].

### Participants

Children between 0 and 18 years old scheduled for a gastrostomy were invited to the study by a surgeon at the participating centers. Exclusions were made if all follow-up questionnaires were missing, as well as if the family were not able to adhere to the follow-up.

The database derived included in total 681 patients, of whom 354 (52,0%) underwent laparoscopic surgery. At least one questionnaire from the three-months follow-up was mandatory to proceed with the analysis of complications, and 311 (87.9%) patients from the laparoscopic group met this criterion. The included patients underwent surgery in five different centers. However, in two centers, only one or no patients met the criteria for laparoscopy and three months follow-up, and were therefore excluded from the data set. In total, 310 patients from three different tertiary centers were analyzed. Median age and weight at the time of the operation were 27.0 months (range 1.0–214.0) and 10.9 kg (range 3.2–84.0 kg) respectively. Patient demographics can be found in Table 1.

Information on the indication for gastrostomy was missing for four patients. Also, some responses in the questionnaires were incomplete, leading to missing data. As a result, the reported numbers for different parameters may be lower than the total number of patients followed up.

**Table 1:** Patient demographics

	Method A	Method B	Method C	Global *p*-value	*P*-value: A vs. B	*P*-value: A vs. C	*P*-value: B vs. C
Median age, months (range)	34 (4–210)	26 (3–214)	24 (1–172)	0.033*	0.682	0.015*	0.062
Median weight, kg (range)	12 (4–84)	11 (4–41)	10 (3–37)	0.020*	0.471	0.006*	0.103
Underlying neurologic disease, number (%)	46/111 (41.4%)	39/70 (55.7%)	70/125 (56.0%)	0.052	0.085	0.036*	1.000

The Kruskal-Wallis test, followed by pair wise Mann-Whitney test was used when comparing age and weight. The Chi-square test was used when comparing underlying neurologic disease. Values are presented as mean (standard deviation) for continuous variables and number (percentage) for categorical variables. Significant *p*-values (< 0.05) are marked in bold and *

### Description of the different laparoscopically assisted techniques

In all techniques a 5 mm laparoscopic trocar was placed sub umbilically with open technique and a 30-degree telescope was used.

**Table 2:** Description of operative techniques

Method	Descriptive term	Method description
Method A	Push-technique with intracorporeal Double U-Stitch	- Abdominal wall thickness measured by syringe needle. (If stomach wall > 20 mm, the technique is replaced by introducer kit and T-fastener with laparoscopic assistance, hence not included in the study.) - 5 mm skin incision and a 5 mm port placed in left upper quadrant at the designated site for the gastrostomy. - Grasp of stomach under laparoscopic visualization. - Attaching the stomach to the anterior abdominal wall with a double U-stitch. The stitch is placed intracorporeally under laparoscopic visualization, starting 0.5–1 cm cranially of the stoma, through the abdominal wall and stomach and back through the abdominal wall 0.5–1 cm caudally of the stoma. Then vice versa on the other side of the grasper. Small skin incisions are made cranially and caudally of the gastrostoma to hide the suture and knot (Polyglactin, Vicryl 3 − 0, CT-1 Plus, Ethicon Inc.). - The stomach wall is externalized and temporary stay sutures and tweezers are used to form a diamond shaped area of the stomach wall, which is incised by scalpel, blade 11. Dilatation of fascia with forceps if needed. - 14 Fr (French) gastrostomy placed, gastroscopic verification of balloon inside the stomach, U-stitch tied with the knot in subcutis.
Method B	Mini-Laparotomy with Fascial- and Purse string sutures	- Small mini-laparotomy incision (at least 10 mm) at designated site for gastrostomy, desufflation. - Four quadrant fascial tacking in posterior fascia, threads used later to tighten the incision to enable insufflation without leakage. (Polydioxanone, PDS 4 − 0, Ethicon Inc.) - Laparoscopic grasp of stomach, desufflation, temporary suture in stomach. - The stomach wall is externalized, placement of one (or two) purse string sutures in the stomach wall. (Poliglecaprone, Monocryl 4 − 0, Ethicon) - The four fascial sutures are secured to the stomach. - Stomach wall opened using diathermy, canal length measured by device and a 12 or 14 Fr gastrostomy placed under direct visualization. - Laparoscopic control performed. - The size of the gastrostoma is reduced in some cases by interrupted resorbable sutures. (Poliglecaprone, Monocryl, Ethicon).
Method C	Push technique with extracorporeal double U-stitch combined with purse-string suture	- 5 mm skin incision and a 5 mm port placed in left upper quadrant at designated site for the gastrostomy. - Grasp of stomach, desufflation. - The stomach wall is externalized, using small fascial incisions when needed. - Double U- stitch/purse string suture through stomach wall and then tunneled under the posterior fascia to attach stomach to the abdominal wall. Small help incisions medially and laterally of the gastrostoma to hide the suture and knot. (Poliglecaprone, BioSyn 3 − 0, GS-21, Covidien, large needle) - Stomach wall opened by scissors and 12 Fr gastrostomy placed, U-stitch/purse string suture tied with knot in subcutis. - Gastroscopic verification of balloon inside the stomach.

### Statistical analysis

The R version 4.4.2 (R Core Team (2024), R Foundation for Statistical Computing, Vienna, Austria) was used for statistical analysis. Kruskal Wallis, Mann-Whitney and chi-square tests were used where appropriate. A sensitivity analysis was performed using logistic regression. *P-*values equal or less than 0.05 were considered significant.

## Results

### Complications in general

**Fig. 1:**
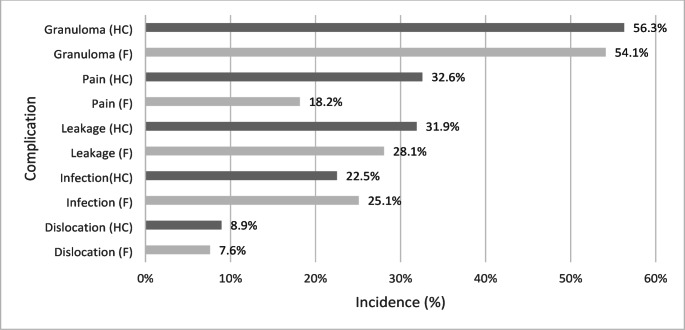
Complications in general at three months postoperatively. Complications reported by health care staff (HC) and families (F) three months after laparoscopic gastrostomy

Health care staff reported some sort of complications in 68.9% and the families in 66.3% during the first three months. Some patients were affected by several different complications (Fig. 1).

### Complications in relation to operative method

The reported incidence of granuloma was significantly higher in method B compared to both method A and C. The complication rates for pain, leakage, infection and granuloma was lower in method C compared to A and B. Dislocation rate did not differ between the methods (see Table 3).

**Table 3:** Postoperative complications during the first 3 months after gastrostomy placement, reported by health care staff and families

Complication	Method A	Method B	Method C	Global *p*-value	*P*-value: A vs. B	*P*-value: A vs. C	*P*-value: B vs. C
Granuloma (HC)	64/108 (59.3%)	61/68 (89.7%)	44/125 (35.2%)	< 0.001*	< 0.001*	< 0.001*	< 0.001*
Granuloma (F)	59/110 (53.6%)	61/68 (89.7%)	44/125 (35.2%)	< 0.001*	< 0.001*	0.007*	< 0.001*
Pain (HC)	44/107 (41.1%)	28/69 (40.6%)	26/125 (20.8%)	0.001*	1.000	0.001*	0.006*
Pain (F)	30/110 (27.3%)	17/68 (25.0%)	8/125 (6.4%)	< 0.001*	0.873	< 0.001*	< 0.001*
Leakage (HC)	50/107 (46.7%)	30/69 (43.5%)	16/125 (12.8%)	< 0.001*	0.789	< 0.001*	< 0.001*
Leakage (F)	39/110 (35.5%)	34/68 (50.0%)	12/125 (9.6%)	< 0.001*	0.078	< 0.001*	< 0.001*
Infection (HC)	35/108 (32.4%)	24/69 (34.8%)	9/125 (7.2%)	< 0.001*	0.870	< 0.001*	< 0.001*
Infection (F)	38/110 (34.5%)	25/68 (36.8%)	13/125 (10.4%)	< 0.001*	0.889	< 0.001*	< 0.001*
Dislocation (HC)	11/108 (10.2%)	6/69 (8.7%)	10/125 (8.0%)	0.841			
Dislocation (F)	10/110 (9.1%)	8/68 (11.8%)	5/125 (4.0%)	0.114			

The Chi-square test was used. First, all three methods were compared. If the global *p-*value was significant, a pairwise test followed. Significant *p-*values (< 0.05) are marked in bold and *

*F* Family, *HC* Healthcare staff

### Sensitivity analysis

Since Method C had a lower incidence of complications, it was designated as the reference category in our logistic regression analysis, which adjusted for age, weight, and neurological disease. The results reveal that the continuous variables (age and weight) were generally non-significant—except for family-reported pain, which was lower among younger children and those with lower body weight. Significant differences were observed between Method C and Methods A and B for the same complications identified in our previous analysis. Furthermore, both healthcare staff and family reported a lower incidence of infection among patients with underlying neurological disease (see Table 4).

**Table 4:** Sensitivity analysis

Complication	Term	Health care staff			Family		
		OR	95%-CI	p-value	OR	95%-CI	p-value
Granuloma	Metod: A (ref: C)	2.92	(1.67–5.19)	< 0.001*	2.16	(1.25–3.78)	0.006*
	Metod: B (ref: C)	20.04	(8.48–55.97)	< 0.001*	16.10	(7.12–41.72)	< 0.001*
	Age	1.00	(0.99–1.01)	0.523	1.00	(0.99–1.01)	0.745
	Weight	1.00	(0.94–1.06)	0.969	1.01	(0.97–1.06)	0.631
	Neurol. disease (ref: No)	1.57	(0.93–2.70)	0.095	1.55	(0.93–2.61)	0.094
Pain	Metod: A (ref: C)	2.49	(1.37–4.59)	0.003*	4.76	(2.08–11.96)	< 0.001*
	Metod: B (ref: C)	2.81	(1.46–5.45)	0.002*	5.37	(2.20–14.15)	< 0.001*
	Age	0.99	(0.98–1.00)	0.150	0.98	(0.97–1.00)	0.036*
	Weight	1.05	(0.99–1.11)	0.093	1.09	(1.03–1.18)	0.012*
	Neurol. disease (ref: No)	0.91	(0.54–1.53)	0.725	0.60	(0.31–1.16)	0.132
Leakage	Metod: A (ref: C)	5.59	(2.91–11.17)	< 0.001*	4.93	(2.42–10.63)	< 0.001*
	Metod: B (ref: C)	5.74	(2.84–12.03)	< 0.001*	10.39	(4.92–23.30)	< 0.001*
	Age	1.00	(0.99–1.01)	0.604	1.00	(0.99–1.01)	0.556
	Weight	1.00	(0.95–1.06)	0.956	1.00	(0.95–1.04)	0.956
	Neurol. disease (ref: No)	0.75	(0.43–1.28)	0.287	0.83	(0.47–1.45)	0.515
Infection	Metod: A (ref: C)	6.42	(2.93–15.36)	< 0.001*	4.37	(2.17–9.25)	< 0.001*
	Metod: B (ref: C)	8.22	(3.57–20.53)	< 0.001*	5.39	(2.52–11.99)	< 0.001*
	Age	0.99	(0.98–1.01)	0.283	1.00	(0.99–1.01)	0.966
	Weight	1.01	(0.95–1.08)	0.720	1.00	(0.96–1.04)	0.857
	Neurol. disease (ref: No)	0.39	(0.21–0.72)	0.003*	0.45	(0.25–0.79)	0.006*
Dislocation	Metod: A (ref: C)	1.35	(0.52–3.51)	0.531	2.48	(0.79–8.57)	0.127
	Metod: B (ref: C)	1.18	(0.38–3.36)	0.765	3.88	(1.22–13.53)	0.024
	Age	0.98	(0.96–1.00)	0.060	0.98	(0.96–1.00)	0.083
	Weight	1.10	(1.00–1.21)	0.058	1.00	(0.88–1.06)	0.950
	Neurol. disease (ref: No)	1.59	(0.69–3.82)	0.281	1.47	(0.59–3.78)	0.414

Sensitivity analysis by logistic regression on reported complications from health care staff and families, using Method C as reference method

*CI* Confidence interval, *OR* Odds ratio

## Discussion

This paper describes three laparoscopically assisted gastrostomy placement techniques used in children at three different tertiary pediatric surgical referral centers in Sweden. It presents differences in complication rates three months postoperatively. The incidence of granuloma was higher in Method B compared to the other methods. We propose that this may be partly due to the larger skin incision, the abundant use of suture material with prolonged resorption times and the presence of residual sutures within the wound area. The incidence of granuloma, pain, infection and leakage was lower in Method C compared to the other methods. This Push-technique involves a small skin incision, and leaves only one rapidly resorbable monofilament suture positioned away from the wound area.

Patient demographics differed between Method A and Method C. Patients in Method C were younger, had lower body weights, and were more likely to have an underlying neurologic disease. Notably, most children referred for gastrostomy have an underlying neurologic disorder and our study’s prevalence of 50.3% aligns well with previous reports ranging from 21.0% to 55.3% [5, 6, 13, 26, 28]. Although some studies have reported an increased risk of superficial surgical site infection and leakage after gastrostomy placement in these patients [8, 29], our findings, in conjunction with other studies [13, 17], do not support this outcome.

A few previously published studies provide relevant comparisons to our results. However, achieving comparable groups across studies is challenging due to variations in surgical methods, time to follow-up and complication assessment.

Sutherland et al. aimed to standardize their gastrostomy technique and conducted a retrospective single-center study to compare seven operative methods, categorized into three groups: Pull-PEG, Push techniques and fascial techniques. They concluded that Push techniques were associated with the lowest complication rates, and that granulomas and leakage were more common when using a fascial suture technique, including open surgery [30]. In our study, we excluded the open technique since it is not the first choice of operative technique at any center.

Kvasnovsky et al. compared two laparoscopic techniques: a modified open technique with fascial sutures and a Seldinger technique, finding that the modified open technique resulted in a higher leakage rate [24]. That partially aligns with our results when comparing Method B and Method C. However, there was no significant difference in leakage rates between Method A and Method B.

Davidson et al. reported a sevenfold increase in granulation tissue in open gastrostomy (which involved a 2 cm skin incision, facial sutures, double purse-string sutures, layered closure and Prolene fixation for one week) compared to laparoscopic Pull-PEG without fixation [28]. This suggest that a larger skin incision and greater amount of suture material may contribute to increased granulation tissue formation.

Naji et al. compared two laparoscopic methods: one using facial sutures and another utilizing a U-stich with introducer kit dilation of the stoma. They found that the modified U-stitch technique was associated with a lower incidence of leakage and dislocation. The authors attributed these improved outcomes to the smaller skin incision, absence of stomach externalization, and minimal handling of gastric tissue [21].

Studies on different suture placement techniques and their associated complication rates show varying results in the literature. Mason et al. compared subcutaneous sutures with external temporary sutures (left in place for 24–48 h) and found no significant differences in minor complications, such as infection or stich abscess. However, they observed an increased risk of complications such as dislocation, readmission to hospital and reoperation in the subcutaneous group [25]. Poola et al. reported ha higher incidence of infections when using subcutaneous sutures compared to temporary fixation for five days [31]. In contrast, Petrosyan et al. found that tunneled subcutaneous fixation was associated with lower complication rates of infection and dislocation, compared to external temporary fixation for three to five days [26].

The type of suture material may also influence the incidence of infection. One study reported that poliglecaprone monofilament (i.e. Monocryl, Ethicon Inc.) was associated with lower complication rates compared to polyglactin braided (i.e. Vicryl Ethicon Inc.) or polydioxanone monofilament (i.e. PDS Ethicon Inc.) [32]. In our study, the incidence of infection was lower in Method C, which utilized poliglecaprone monofilament (BioSyn, Covidien). Methods A and B had comparable infection rates using polyglactin braided (Vicryl, Ethicon) respective polydioxanone monofilament (i.e. PDS Ethicon Inc.) and poliglecaprone monofilament (i.e. Monocryl, Ethicon Inc.).

## Strengths and limitations

The relatively large number of patients, encompassing most pediatric gastrostomies performed Sweden, ensures that our data is a reliable source. Additionally, the prospective design assures that all required information is available. Since pediatric health care in Sweden is free of charge for the families, it is unlikely that dropout rates were influenced by socio-economic factors.

This study has several limitations. First, its observational design may introduce biases that limit causal inferences. Additionally, because the surgeries were performed across multiple centers by different surgeons, there is potential variability in operative techniques that could affect the outcome.

The assessment of complications may also vary as follow-up questionnaires were completed by several different nutritional nurses, and in some cases a physician. To reduce bias, the health care staff received written material on how to report complications and the family’s perspective were included. In most cases the family’s responses corresponded well with those of the healthcare staff. However, discrepancies in reported postoperative pain were noted between healthcare staff and families. We believe that families tend to report less pain, as they have a deeper understanding of their child´s expression of pain, distinguishing them better from anxiety or fear. Furthermore, families evaluate pain over the entire postoperative period, whereas health care staff assess pain only during the first gastrostomy button replacement, a situation where both fear and pain may be present and difficult to differentiate. Since most patients in this study are unable to communicate pain themselves, our findings rely on the family’s perception of pain.

Our previously published study, on the same data set, revealed a reduction in complications (granuloma, pain, leakage and infection) when using a 12 Fr (French) tube compared to a 14 Fr tube. In Method A, 14 Fr tubes were used exclusively, while in Method C 12 Fr were used. In Method B, 12 Fr were used in 37,5% of patients. We also found that using a tube with an additional 2 mm of length relative to the gastrostomy canal, was associated with fewer postoperative complications compared to other tube lengths. The additional length of 2 mm was used in 36.2%, 22.7% and 100% of the cases for Method A, B and C respectively [17]. Upon reviewing the operative methods, we realized that the Methods A and B employed a more reliable measurement technique compared to Method C, where the distance was more of an estimation by the surgeon.

The advantage of a 12 Fr tube may influence our result, and may partly explain the differences in complications seen in method A compared to C, where the operation technique is very similar to each other. A prospective randomized study on 12 or 14 Fr tube could clarify that, and to our knowledge that has not been done yet. A couple of studies comparing 12 Fr and 14 Fr tubes suggest that a 14 Fr tube has significantly lower rates of complications such as dislocation and obstruction, but did not include other complications [33, 34]. However, in our previously published study the dislocation rates were similar when using 12 Fr or 14 Fr tube [17].

## Conclusion

Approximately two-thirds of children undergoing gastrostomy are affected by at least one minor postoperative complication, with granuloma being the most common. These complications not only affect the quality of life of this already vulnerable patient population, but also place a burden on the healthcare system, emphasizing the need for preventive strategies. Granulation tissue formation may result from irritation caused by moisture from leakage, gastrostomy mobility, or tissue inflammation due to residual suture material at the wound site. To minimize these risks, we recommend using a small skin incision and limiting the use of suture material, ensuring it is placed away from the gastrostomy site.

## Data Availability

The data that support the findings of this study are available from the corresponding author upon reasonable request. Data are located in controlled access data storage at Akademiska sjukhuset, Uppsala.

## Abbreviations

CI: Confidence interval
F: Family
Fr: French
HC: Health care staff
OR: Odds ratio
PEG: Percutaneous endoscopic gastrostomy
